# Supplementary material for: The Impact of the COVID-19 Pandemic on Burnout, Compassion Fatigue, and Compassion Satisfaction in Healthcare Personnel: A Systematic Review of the Literature Published during the First Year of the Pandemic
Source: Healthcare (Basel). 2022 Feb 13;10(2):364. doi: 10.3390/healthcare10020364 (PMC8872521; doi:10.3390/healthcare10020364)
Supplement: Supplementary file 1 [file healthcare-10-00364-s001.zip › healthcare-1584789 sup.pdf]

Supplement S1. PRISMA CHECKLIST

Section/topic	#	Checklist item	Reported on page #
**TITLE**			
Title	1	Identify the report as a systematic review, meta-analysis, or both.	1
**ABSTRACT**			
Structured summary	2	Provide a structured summary including, as applicable: background; objectives; data sources; study eligibility criteria, participants, and interventions; study appraisal and synthesis methods; results; limitations; conclusions and implications of key findings; systematic review registration number.	1
**INTRODUCTION**			
Rationale	3	Describe the rationale for the review in the context of what is already known.	2
Objectives	4	Provide an explicit statement of questions being addressed with reference to participants, interventions, comparisons, outcomes, and study design (PICOS).	2-3
**METHODS**			
Protocol and registration	5	Indicate if a review protocol exists, if and where it can be accessed (e.g., Web address), and, if available, provide registration information including registration number.	3
Eligibility criteria	6	Specify study characteristics (e.g., PICOS, length of follow-up) and report characteristics (e.g., years considered, language, publication status) used as criteria for eligibility, giving rationale.	3
Information sources	7	Describe all information sources (e.g., databases with dates of coverage, contact with study authors to identify additional studies) in the search and date last searched.	3-4
Search	8	Present full electronic search strategy for at least one database, including any limits used, such that it could be repeated.	3-4

Section/topic	#	Checklist item	Reported on page #
Study selection	9	State the process for selecting studies (i.e., screening, eligibility, included in systematic review, and, if applicable, included in the meta-analysis).	3
Data collection process	10	Describe method of data extraction from reports (e.g., piloted forms, independently, in duplicate) and any processes for obtaining and confirming data from investigators.	4
Data items	11	List and define all variables for which data were sought (e.g., PICOS, funding sources) and any assumptions and simplifications made.	3-4
Risk of bias in individual studies	12	Describe methods used for assessing risk of bias of individual studies (including specification of whether this was done at the study or outcome level), and how this information is to be used in any data synthesis.	Not applicable
Summary measures	13	State the principal summary measures (e.g., risk ratio, difference in means).	4
Synthesis of results	14	Describe the methods of handling data and combining results of studies, if done, including measures of consistency (e.g., I^2) for each meta-analysis.	Not applicable
Risk of bias across studies	15	Specify any assessment of risk of bias that may affect the cumulative evidence (e.g., publication bias, selective reporting within studies).	Not applicable
Additional analyses	16	Describe methods of additional analyses (e.g., sensitivity or subgroup analyses, meta-regression), if done, indicating which were pre-specified.	Not applicable
**RESULTS**			
Study selection	17	Give numbers of studies screened, assessed for eligibility, and included in the review, with reasons for exclusions at each stage, ideally with a flow diagram.	4
Study characteristics	18	For each study, present characteristics for which data were extracted (e.g., study size, PICOS, follow-up period) and provide the citations.	50
Risk of bias within studies	19	Present data on risk of bias of each study and, if available, any outcome level assessment (see item 12).	Not applicable

Section/topic	#	Checklist item	Reported on page #
Results of individual studies	20	For all outcomes considered (benefits or harms), present, for each study: (a) simple summary data for each intervention group (b) effect estimates and confidence intervals, ideally with a forest plot.	Not applicable
Synthesis of results	21	Present results of each meta-analysis done, including confidence intervals and measures of consistency.	Not applicable
Risk of bias across studies	22	Present results of any assessment of risk of bias across studies (see Item 15).	Not applicable
Additional analysis	23	Give results of additional analyses, if done (e.g., sensitivity or subgroup analyses, meta-regression [see Item 16]).	Not applicable
**DISCUSSION**			
Summary of evidence	24	Summarize the main findings including the strength of evidence for each main outcome; consider their relevance to key groups (e.g., healthcare providers, users, and policy makers).	52
Limitations	25	Discuss limitations at study and outcome level (e.g., risk of bias), and at review-level (e.g., incomplete retrieval of identified research, reporting bias).	52
Conclusions	26	Provide a general interpretation of the results in the context of other evidence, and implications for future research.	53
**FUNDING**			
Funding	27	Describe sources of funding for the systematic review and other support (e.g., supply of data); role of funders for the systematic review.	Not applicable

Supplement S2. Keywords and search terms used in the systematic review

Regarding the first terms of the search, Pandemic or COVID-19 or SARS-COV-2 or Coronavirus, the thesauri for them included in the *Medical Subject Headings* (MeSH) and that were included as keywords, were:

- Pandemics – no thesauri.
- COVID-19, the following thesauri are found:
    - 2019 novel coronavirus disease
    - COVID19
    - COVID-19 pandemic
    - SARS-CoV-2 infection
    - COVID-19 virus disease
    - 2019 novel coronavirus infection
    - 2019-nCoV infection
    - coronavirus disease 2019
    - coronavirus disease-19
    - 2019-nCoV disease
    - COVID-19 virus infection
- SARS-COV-2 – no thesauri.
- Coronavirus, only the following thesaurus is used:
    - Coronaviruses

As for the second search terms, Burnout or Compassion Fatigue or Stress Disorders or Compassion Satisfaction, the thesauri for them included in the *Medical Subject Headings* (MeSH) and included as keywords were:

- Compassion Fatigue, the following thesauri are found:
    - Fatigue, Compassion
    - Vicarious Trauma
    - Trauma, Vicarious
    - Traumas, Vicarious
    - Vicarious Traumas
    - Secondary Trauma
    - Secondary Traumas
    - Trauma, Secondary
    - Traumas, Secondary
    - Secondary Traumatization

    - Secondary Traumatizations
    - Traumatization, Secondary
    - Traumatizations, Secondary
    - Secondary Traumatic Stress
    - Stress, Secondary Traumatic
    - Stresses, Secondary Traumatic
    - Traumatic Stress, Secondary
    - Vicarious Traumatization
    - Traumatization, Vicarious
- Burnout, Professional, the following thesauri are found:
    - Professional Burnout
    - Occupational Burnout
    - Burnout, Occupational
    - Career Burnout
    - Burnout, Career
- Stress Disorders, Post-Traumatic, the following thesauri are found:
    - PTSD
    - Stress Disorder, Post Traumatic
    - Neuroses, Posttraumatic
    - Posttraumatic Neuroses
    - Posttraumatic Stress Disorders
    - Posttraumatic Stress Disorder
    - Stress Disorder, Posttraumatic
    - Stress Disorders, Posttraumatic
    - Neuroses, Post-Traumatic
    - Neuroses, Post Traumatic
    - Post-Traumatic Neuroses
    - Post-Traumatic Stress Disorders
    - Post Traumatic Stress Disorders
    - Post-Traumatic Stress Disorder
    - Stress Disorder, Post-Traumatic
    - Moral Injury
    - Injury, Moral
    - Moral Injuries

    - Delayed Onset Post-Traumatic Stress Disorder
    - Delayed Onset Post Traumatic Stress Disorder
    - Acute Post-Traumatic Stress Disorder
    - Acute Post Traumatic Stress Disorder
    - Chronic Post-Traumatic Stress Disorder
    - Chronic Post Traumatic Stress Disorder
- Compassion satisfaction – no thesauri.

Finally, regarding the last search terms, Health professionals or Nursing staff or Nurses or Physicians or Psychologists, their thesauri included in the Medical Subject Headings (MeSH) and included as keywords, were:

- Health personnel, the following thesauri are found:
    - Personnel, Health
    - Health Care Providers
    - Health Care Provider
    - Provider, Health Care
    - Providers, Health Care
    - Healthcare Providers
    - Healthcare Provider
    - Provider, Healthcare
    - Providers, Healthcare
    - Healthcare Workers
    - Healthcare Worker
- Nursing staff, the following thesauri are found:
    - Staff, Nursing
    - Staffs, Nursing
    - Nursing Staffs
- Nurses, the following thesauri are found:
    - Nurse
    - Personnel, Nursing
    - Nursing Personnel
    - Registered Nurses
    - Nurse, Registered
    - Nurses, Registered
    - Registered Nurse

- Physicians – no thesauri.
- Psychologists – no thesauri.

Therefore, the equation used in the search strategy was the following:

- ((Pandemics) OR (COVID-19) OR (2019 novel coronavirus disease) OR (COVID19) OR (COVID-19 pandemic) OR (SARS-CoV-2 infection) OR (COVID-19 virus disease) OR (2019 novel coronavirus infection) OR (2019-nCoV infection) OR (coronavirus disease 2019) OR (coronavirus disease-19) OR (2019-nCoV disease) OR (COVID-19 virus infection) OR (SARS-COV-2) OR (Coronavirus) OR (Coronaviruses)) AND
- ((Compassion Fatigue) OR (Fatigue, Compassion) OR (Vicarious Trauma) OR (Trauma, Vicarious) OR (Traumas, Vicarious) OR (Vicarious Traumas) OR (Secondary Trauma) OR (Secondary Traumas) OR (Trauma, Secondary) OR (Traumas, Secondary) OR (Secondary Traumatization) OR (Secondary Traumatizations) OR (Traumatization, Secondary) OR (Traumatizations, Secondary) OR (Secondary Traumatic Stress) OR (Stress, Secondary Traumatic) OR (Stresses, Secondary Traumatic) OR (Traumatic Stress, Secondary) OR (Vicarious Traumatization) OR (Traumatization, Vicarious) OR (Burnout) OR (Burnout, Professional) OR (Professional Burnout) OR (Occupational Burnout) OR (Career Burnout) OR (Burnout, Career) OR (Stress Disorders, Post-Traumatic) OR (PTSD) OR (Stress Disorder, Post Traumatic) OR (Neuroses, Posttraumatic) OR (Posttraumatic Neuroses) OR (Posttraumatic Stress Disorders) OR (Posttraumatic Stress Disorder) OR (Stress Disorder, Posttraumatic) OR (Stress Disorders, Posttraumatic) OR (Neuroses, Post-Traumatic) OR (Neuroses, Post Traumatic) OR (Post-Traumatic Neuroses) OR (Post-Traumatic Stress Disorders) OR (Post Traumatic Stress Disorders) OR (Post-Traumatic Stress Disorder) OR (Stress Disorder, Post-Traumatic) OR (Moral Injury) OR (Injury, Moral) OR (Moral Injuries) OR (Delayed Onset Post-Traumatic Stress Disorder) OR (Delayed Onset Post Traumatic Stress Disorder) OR (Acute Post-Traumatic Stress Disorder) OR (Acute Post Traumatic Stress Disorder) OR (Chronic Post-Traumatic Stress Disorder) OR (Chronic Post Traumatic Stress Disorder) OR (Compassion Satisfaction) OR (Satisfaction, Compassion)) AND
- ((Health personnel) OR (Personnel, Health) OR (Health Care Providers) OR (Health Care Provider) OR (Provider, Health Care) OR (Providers, Health Care) OR (Healthcare Providers) OR (Healthcare Provider) OR (Provider, Healthcare) OR (Providers, Healthcare) OR (Healthcare Workers) OR (Healthcare Worker) OR (Nursing staff) OR (Staff, Nursing) OR (Staffs, Nursing) OR (Nursing Staffs) OR (Nurses) OR (Nurse) OR (Personnel, Nursing) OR (Nursing Personnel) OR (Registered Nurses) OR (Nurse,

Registered) OR (Nurses, Registered) OR (Registered Nurse) OR (Physicians) OR (Psychologists))
